# A Method Based on Differential Entropy-Like Function for Detecting Differentially Expressed Genes Across Multiple Conditions in RNA-Seq Studies

**DOI:** 10.3390/e21030242

**Published:** 2019-03-04

**Authors:** Zhuo Wang, Shuilin Jin, Chiping Zhang

**Affiliations:** Department of Mathematics, Harbin Institute of Technology, Harbin 150006, China

**Keywords:** differential entropy-like function, differential expressed genes, multiple condition data, time-course data

## Abstract

The advancement of high-throughput RNA sequencing has uncovered the profound truth in biology, ranging from the study of differential expressed genes to the identification of different genomic phenotype across multiple conditions. However, lack of biological replicates and low expressed data are still obstacles to measuring differentially expressed genes effectively. We present an algorithm based on differential entropy-like function (DEF) to test for the differential expression across time-course data or multi-sample data with few biological replicates. Compared with limma, edgeR, DESeq2, and baySeq, DEF maintains equivalent or better performance on the real data of two conditions. Moreover, DEF is well suited for predicting the genes that show the greatest differences across multiple conditions such as time-course data and identifies various biologically relevant genes.

## 1. Introduction

Next-generation sequencing (NGS) technology has rapidly become the tool for many genome-wide transcription studies. Production of millions or billions of short sequences from individual RNA molecules together with lower costs and higher budgets have enabled many methodologies, such as RNA sequencing (RNA-Seq) [1]. RNA-Seq technology empowers thorough recognition of gene isoforms [2], translocations [3], nucleotide variations [4], time-course gene expression analysis [5], and cap analysis of gene expression (CAGE) [6]. One reason for the growing popularity of RNA-Seq technology is its ability to detect differentially expressed genes between two or more conditions (e.g., different races of human populations). However, the expression of most genes is intrinsically stochastic [7], so several methods have been introduced for exploring it [8,9,10], despite the variability in RNA-Seq data, which poses challenges for differential expression and other relative analysis [11].

The theoretical methods for the study of RNA-Seq data can be grouped into two major categories. The first representative methods are limma [10], edgeR [12,13,14], DESeq2 [15], baySeq [16], PoissonSeq [17,18], DSS [19] and DGEclust [20]. These methods rely on the accuracy of distribution assumptions and parameters estimation. Once the reads are mapped at the gene, exon or transcript level, the problem is naturally summarized into matrices where rows represent exons, genes or transcripts and columns represent samples or replicates. Therefore, many of the earliest statistical methods are based on Poisson or negative binomial distribution to model read counts. Some of these methods represent the current state-of-the-art of the field for the study of RNA-Seq data. However, the methods based on the negative binomial and Poisson model either fail to perform multiple conditions comparison or are excessively conservative [21,22]. It is noteworthy that assessing differential low expressed count features and biological experiments with few replicates is a challenge for the estimation of model parameters. The methods in the second category such as NOIseq [23,24], rSeqNP [25] and SAMseq [26] attempt to make use of non-parametric approaches. These methods rely less on the probability distribution of exon, gene and transcript counts. An advantage is that this class of methods aims to be data-adaptive and is suitable for differential expression analysis without prior information. Whereas when we teased apart this kind of methods, we found that they may tend to overestimate differentially expressed genes with high variability among replicates.

In this paper, we introduce an approach based on the differential entropy-like function (DEF), an algorithm for discovering the differentially expressed genes across multiple conditions. From the theoretical point of view, the importance of the proposed method derives from its information-theoretic background. On the one hand, DEF allows the recognition of differentially expressed genes on multiple conditions such as time-course data and multiple tissues data. Compared with the popular alternative methods, DEF obtains a wider application. On the other hand, DEF is effective in detecting the differentially expressed genes with the characteristic that it does not rely on the probability distribution. Another characteristic of DEF is its adaptability on zero expressed gene counts, which could give rise to the other troubling aspect that many hypothesis test methods fail. The paper is organized into the following sections. Section 2 presents the performance of DEF compared with the other methods on the datasets of two conditions. Section 3 presents the evaluation of DEF on the two-condition data. Section 4 presents the performance of the DEF on the time-course RNA-Seq data. Section 5 presents the effective analysis of DEF on the datasets of multiple conditions.

## 2. Results

### 2.1. DEF Shares Many Genes with Limma, DESeq2, baySeq and edgeR

**Figure 1:**
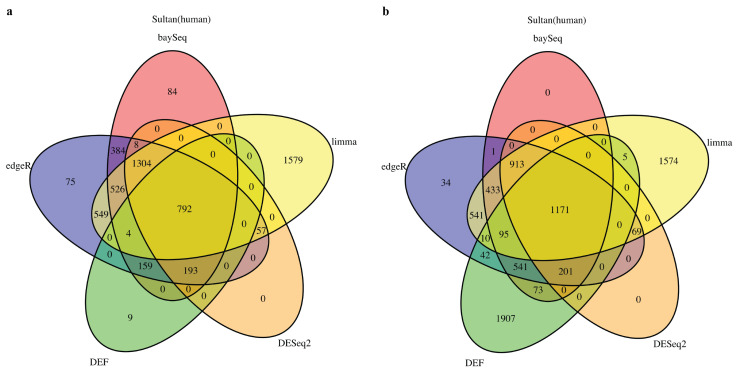
Venn diagram of differentially expressed genes obtained from limma, baySeq, DESeq2, edgeR and DEF: (**a**) read counts from “Sultan” dataset with the threshold for DEF entropy-like value of 0.05; and (**b**) read counts from “Sultan” dataset with the threshold for DEF entropy-like value of 0.01.

**Figure 2:**
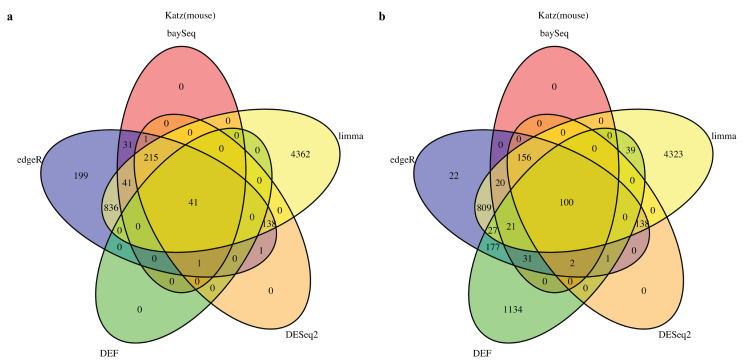
Venn diagram of differentially expressed genes obtained from limma, baySeq, DESeq2, edgeR and DEF: (**a**) read counts from “Katz” dataset with the threshold for DEF value of 0.05; and (**b**) read counts from “Katz” dataset with the threshold for DEF value of 0.01.

We started by analyzing the actual RNA-Seq datasets with limma, DESeq2, baySeq, edgeR and DEF. Details of the datasets are described in Table 8 of the “Materials and Methods” Section. In all cases with “Sultan” and “Katz” datasets from R package “recount” [27], there were two-condition data with two technical replicates per condition. The Venn diagram for each of the cases is shown in Figure 1 and Figure 2. All compared methods ranked each gene by providing *P* values (limma, DESeq2 and edgeR), FDR (baySeq) or an entropy-like value (DEF). *P* Value or FDR less than 0.05 was considered to indicate statistical significance. The cut-off values of DEF were 0.05 (Figure 1a and Figure 2a) and 0.01 (Figure 1b and Figure 2b). As indicated by the Venn diagrams constructed from differentially expressed genes, sharing 792 genes in the “Sultan” case demonstrated a significant overlap by the five methods (Figure 1a). In this figure, we note that the differentially expressed genes found by DEF were to a large extent also found by limma, edgeR, baySeq and DESeq2. Simultaneously, our DEF method found a fair amount of unique differentially expressed genes, which were not shared with the other methods (Figure 1b). We further investigated two possibilities for the additional differentially expressed genes in Figure 1b. Table 1 and Table 2 list the raw read counts of ten unique differentially expressed genes detected by DEF (Figure 1b). The top five and last five genes were with the largest and smallest DEF values among the 1907 additional genes. As can be seen in Table 1, all five genes had only one non-zero expressed value across four replicates. These genes were true positives, which DEF detected better than the other methods. As shown in Table 2, the differences between these replicates were not so clear. Some gene such as “ENSG00000065357” had extreme read counts (10), while some genes had moderate read counts. These genes could be false positives detect by DEF, which was why the other methods did not identify them. In “Katz” case, 41 and 100 overlapping genes were found by each method with different cut-off values of DEF (Figure 2). Figure 2b displays that DEF detected 1134 additional differentially expressed genes. Table 3 and Table 4 demonstrate the raw read counts of “Katz” dataset, which further confirms the effectiveness of DEF method. The two tables list the top five and last five unique differentially expressed detected by DEF from 1134 genes (Figure 2b). In Table 3, five genes with largest DEF values expressed in Condition B. However, they all had zero expressed values in Condition A. These genes should be different genes and DEF successfully detected these genes, which failed to be identified by the other methods. In Table 4, the gene “ENSMUSG00000036977” was highly expressed in Condition A and the genes “ENSMUSG00000057924” and “ENSMUSG00000067203” had higher expression in Replicate 1 of Condition B. These genes were true positives detected by DEF. The difference performance of the other genes was not clear. These genes could be false positives.

The reasons for better performance of DEF compared to other methods in all datasets stem from its adaptability for zero expressed gene counts. Low replicates in each sample cause inaccuracy on the distribution-dependent methods and zero expressed gene counts give rise to the other troubling aspect that many hypothesis test methods failed.

### 2.2. DEF Successfully Identified the Differentially Expressed Genes under the Real Dataset

**Figure 3:**
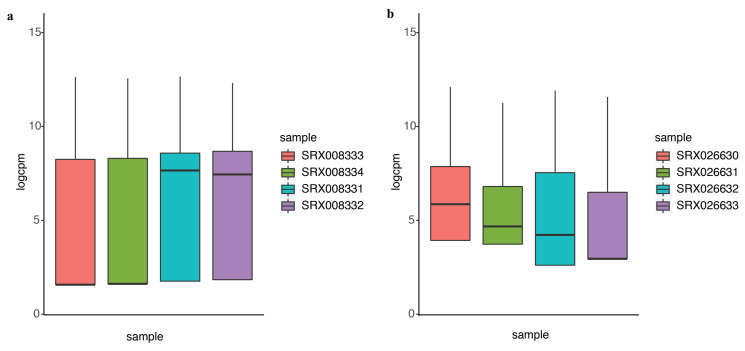
The performance of DEF on the two-group data: (**a**) box plots of the top 100 differentially expressed genes from “Sultan” dataset; and (**b**) box plots of the top 100 differentially expressed genes from “Katz” dataset.

In differential expression analysis, an important task is to identify the genes that are differentially expressed at higher variability between experimental conditions without prior information. We compared the performance of DEF to identify differentially expressed genes under the experimental conditions encapsulated by the actual dataset. Details of the dataset are in the “Materials and Methods” Section. We analyzed the 100 top-ranking genes with box plots, as shown in Figure 3a for “Sultan” dataset and Figure 3b for “Katz” dataset. These box plots show the variance across different samples. Medians of the box plots varied widely across samples. Hence, DEF is an approach for the identification of differentially expressed genes from count data. Essentially, our method creates a measurement for gene-wise counts by DEF and evaluates the absolute expression differences for the genes in all the samples. We also utilized the method generalized log-cpm to obtain the normalized matrix of counts, which can remove potentially library variation and prevent bias and mean squared error in downstream analyses. We evaluated various methods for differential expression analysis and found that our method performed equivalent to the classical methods. The main difference between DEF method and other methods based on statistics is the ability to handle low expression counts, especially zero counts, an issue of great importance when investigating differential expression in the context of RNA-Seq. When both samples have zero reads, clearly nothing can be said about differential expression and we have already filtered these genes. Presumably, this represents an interesting biological phenomenon, where a gene in all samples is completely non-expressed according to sequencing. For genes with zero counts in either sample, many methods failed, except DEF method (Table 1 and Table 3). Because many methods cannot handle zero-count genes, their methods failed to detect many easy cases of differentially expressed genes (i.e., genes with zero-count in one condition while non-zero-count in the other condition). Because the use of parameters in the normalization step of our model successfully handled zero counts, the larger DEF value was particularly pronounced for genes expressed at a more obvious difference across samples (Table 1, Table 2, Table 3 and Table 4).

### 2.3. DEF Is Applicable for the Real Time-Course Data

**Figure 4:**
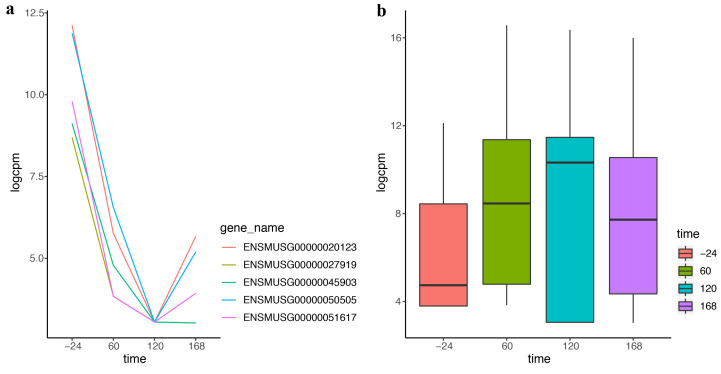
The performance of DEF on time-course data: (**a**) normalized read counts of the top five differentially expressed genes over four time points; and (**b**) box plots of the top 100 differentially expressed genes on the first time point compared with every time point.

With the development of sequencing technology, researchers pay more attention to timeliness. More and more time-course data come from the advanced experiment and more methods are needed for analyzing these data. DEF also works effectively on the time-course data. “Trappnell” dataset was obtained from online resources of “recount”. The mouse samples of the dataset were from four time points following differentiation. We applied DEF on the time-course data and took the top five differentially expressed genes with the largest DEF values for further analysis. Figure 4a displays dramatic changes over the four time points of the top five differentially expressed genes. The genes decreased rapidly in the first two time courses and increased in the last time course. These genes could be used for further analysis in the biological progress. Table 5 lists the gene symbols and gene functions of these five genes. The most differentially expressed gene, Kart9, plays an essential role in the correct development of sperm [28]. The Lce1g is one of the differentiation-related genes [29]. All these findings were consistent with the characteristic of the time-course data. Particularly, our results predicted that Avpr1a, Pcdh20 and Npas4 may play an important role in the shaping of the samples from time-course data. Figure 4b shows the box plot of the top 100 differentially expressed genes of the four time points separately. Comparison of the four box plots clearly indicates the expression variation across four time points, which proved the effectiveness of DEF.

### 2.4. DEF Is Applicable for the Real Multiple Condition Data

**Figure 5:**
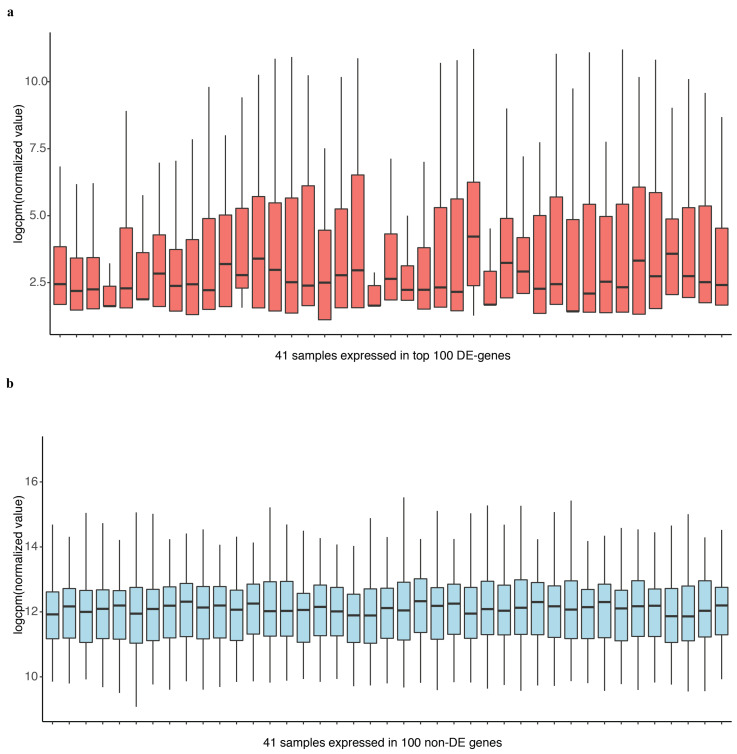
Box plot of the top 100 differentially expressed genes and last 100 non DE genes of 41 samples separately.

**Figure 6:**
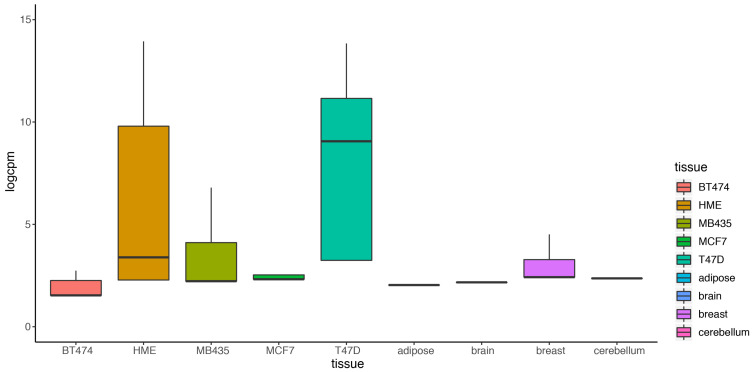
Box plot of the top 100 differentially expressed of nine tissues separately.

Most existing methods deal with a two-condition comparison, while DEF was designed to be an effective tool for the quantification of differentially expressed genes across multiple conditions. We show that our method is applicable in terms of differential expression analysis on multiple condition data. A multiple condition dataset was considered as an example to test the feasibility of our method. We chose the “Cheung” dataset, which contains samples of 41 CEPH HapMap (CEU) samples [30], and applied DEF method for differential expression analysis across the 41 samples. DEF identified several genes that are differentially expressed across these samples (Table 6). We searched for some testified findings and these findings were consistent with our results. Our observation about the differentially expressed genes implicated that DEF is well suited for predicting which genes show the greatest differences in expression between biological samples. One of the differentially expressed genes is ZFP57, which is confirmed as highly variably expressed gene from 1000 Genomes CEU phase 1 [31]. The “Cheng” dataset consists of 17 female samples and 24 male samples. Another gene RPS4Y1 is also a differentially expressed gene detected by DEF. The gene RPS4Y1 is located in chromosome Y. In particular, our results predict that PRSS21, MKRN and GTSF1 may play an important role in the shaping of specificity of the CEU from HapMap (Table 6). Figure 5a shows the box plot of the top 100 differentially expressed genes across all the samples separately. Figure 5b displays 100 genes that DEF identified as non-differentially expressed genes. Clearly, medians change greatly in Figure 5a while the medians in Figure 5b are robust. Comparison of 41 box plots in Figure 5a,b clearly indicates the expression variation across samples, which proved the effectiveness of DEF. We also took additional multi-tissue data as an example to test the feasibility of our method. We chose the data “Wang” from online resources “recount” of RNA sequencing on human cell line with diverse tissues. The tissues included in the dataset are cerebellum, breast, brain, adipose, T47D, MCF7, MB435, HME and BT474. We compared the dataset from these tissues and applied DEF method for differential expression analysis between those samples. As there are limited “gold-standard” data with which to evaluate the accuracy of RNA-Seq quantification methods, and because real differentially expressed genes are difficult to confirm, we connected some gene functions of our analyzed DE genes with the real biological differential traits between different tissues. TCL1A [32,33], POU2AF1, ARHGDIB [34,35,36], LRMP and IRS4 [37,38,39] were the most variable genes (Table 7).We also took the top 100 differentially expressed genes of every sample for further analysis. The result presented in Figure 6 show that our method found the obvious differences across the different samples. We found that there is limited “gold-standard” data with which to evaluate the accuracy of RNA-Seq quantification methods, calling into question how to thoroughly evaluate the DEF method. However some published findings ar e consistent with our results.

## 3. Discussion

In this study, we performed a detailed comparative analysis of DEF method with several other methods for differential expression analysis by RNA-Seq data. For these methods, we focused on the specialty of generalized log-cpm normalization, especially conditions on low expression read counts. Small sample statistical analysis is still a tough task, while our proposed method could avoid the bias to some extent. Low biological or technical replicates in each sample could cause inaccuracy on the distribution-dependent methods. In contrast to other approaches, our model implies that the numbers of read counts are not entirely distinct, but they are connected between samples of replicates. This is a form of information sharing between genes and between samples, which are made possible by calculating the differential entropy-like function of the proposed model. The important contribution of this study is the solution of zero counts when performing differential expression analysis. Zero expressed gene counts give rise to the further troubling aspect that many hypothesis test methods fail. However, our model successfully handles the two dilemmas. We emphasize that the difference does exist when one sample has zero read counts while the other does not. DEF not only shares many differentially expressed genes but also detects additional differentially expressed genes. Additionally, we studied the effect of the standard deviation on the gene-wise mean expression level. DEF is also faster and more convenient, and converts RNA-Seq data into a form that can be analyzed using a single value of a gene. It is demonstrated that combining generalized log-cpm normalization with provided DEF function can lead to a more powerful analysis to other alternatives, such as either parameter-dependent method or some other distribution-dependent method on their own. The last and the most meaningful contribution of our DEF method is its application on variable biological conditions with multi-sample and time-course data. The biological basis for diversity in gene expression between different conditions is likely to be complex. Analysis of multiple samples and time-course data helps to expose the biological bases underlying tissue diversity. The analysis of data from the same or different populations studies that each contained some population replicates showed us that the differentially expressed genes made by our strategy were likely to be biologically meaningful, as their phenotypes do have to distinguish among the gene set we test.

## 4. Conclusions

DEF performed as well as existing RNA-Seq methods, especially when the gene-wise expression was low. Meanwhile, DEF performed effectively on multiple conditions such as multi-sample and time-course RNA-Seq data. One characteristic of DEF is its independence of the statistical distribution of feature counts. Most methods for gene expression differential analysis are based on the negative binomial model, which seems unreasonable sometimes. Low replicates in each group cause inaccuracy in the distribution-dependent methods and zero expressed gene counts give rise to the other troubling aspect that many hypothesis test methods fail. However, DEF based on information theory successfully deals with the two issues. Following the previous studies, in differential expression analysis, an important task is to identify the genes that are expressed at higher variability across multiple samples without prior information. Accumulations of comparative studies for multi-sample data are desired, which is what we have always been focused on. DEF could work well with multiple conditions even with few replicates. The different degree of genes could be ordered by their relative DEF values. Moreover, we expect our work to inspire and support further theoretical research on modeling gene expression data and we believe that our software, DEF, will prove to be a useful addition to the existing methods for the statistical analysis of RNA-Seq and similar types of data. Some cell types will be more similar to each other, which will pose more challenges for meta-data analysis. Nevertheless, we speculate that the current method can be applied to single-cell data and comprehensive evaluations are the subsequent tasks.

## 5. Materials and Methods

### 5.1. Dataset

All datasets were obtained from the “recount” online resource http://bowtie-bio.sourceforge.net/recount/. We used the R package “recount” to get each count table combined with sample phenotype data. Table 8 lists the details of the four datasets analyzed in this article.

### 5.2. Normalization

Normalization of the count data is a crucial step in the analysis of RNA-Seq data, which has a strong impact on the detection of differentially expressed genes. A normalization strategy called generalized log-cpm was used. The log-cpm method is a well-accepted normalization step of gene expression by dividing the corresponding library size (in millions) of each read count shown in voom method [43]. Specifically, the entry point of the normalization algorithm is a set of *n* RNA samples whose sequence reads have been summarized according to the number mapping to each gene. The raw matrix *Y* with elements $y_{ij}\left(i=1,\ldots ,m;j=1,\ldots ,n\right)$ indicates the number of sequencing reads that have been mapped to a gene in a sample. Write $Y_{j}$ for the total number of mapped reads for sample *j*, $Y_{j}=\sum_{j=1}^{n}{\tilde{y}}_{ij}$.

(1) $${\bar{y}}_{ij}=log_{2}\left(\frac{y_{ij}+0.5}{Y_{j}+1}\times 10^{6}\right)$$

where $y_{ij}$ is the number of reads mapped to gene *i* in sample *j*. However, log-cpm values could be negative, which is a problem in some specific conditions. Besides, the normalization size $10^{6}$ was fixed, which may cause the log-cpm value to be too large or too small. To avoid these problems, a generalized log-cpm value was given:

(2) $${\tilde{y}}_{ij}=log_{2}\left(\frac{y_{ij}+1}{Y_{j}+1}\times 10^{k}\right)$$

where $k=[log_{10}\left(\underset{j}{max}Y_{j}\right)]+1$. Note that the positive ${\tilde{y}}_{ij}$ was normalized by the maximal reads of each row. It is worth noting that the denominator counts $Y_{j}$ was offset by one to avoid the meaningless condition when $Y_{j}$ equals zero. At the same time, the numerator counts $y_{ij}$ were augmented by a small positive value (one read) to avoid taking the logarithm of zero. Such operation not only ensures no missing generalized log-cpm values but also allows that $\frac{y_{ij}+1}{Y_{j}+1}$ is less than one as well as greater than zero. To avoid the phenomenon that it is highly possible that ${\tilde{y}}_{ij}$ is negative, the parameter *k* played an important role to ensure that $\frac{y_{ij}+1}{Y_{j}+1}\times 10^{k}$ was strictly greater than one. As a result, ${\tilde{y}}_{ij}$ was strictly greater than zero. Moreover, the benefit of generalized log-cpm is not only dealing with zero expression read counts but also decreasing the variance of the genes with larger RNA-Seq counts. In particular, the generalized log-cpm ensures no missing values and reduces the variability at high expressed count values. In summary, generalized log-cpm method for normalization is crucial in terms of differentiating between gene expression changes with low expressed or extremely high expressed read counts.

### 5.3. DEF Function

Subsequently, a novel method based on the differentially entropy-like function was used for detecting the differentially expressed genes across multiple samples in RNA-Seq data. Internally, DEF uses the normalization method generalized log-counts per million, which is a simple and reasonable scale for normalization by providing scale factors to make counts comparable between different samples. For the normalized read counts matrix $\tilde{Y}$, the differential entropy-like function is defined as

(3) $$H=1-\frac{-\sum_{j=1}^{n}\left[\left(\frac{{\tilde{y}}_{ij}}{Y_{j}}\right)\cdot log\left(\frac{{\tilde{y}}_{ij}}{Y_{j}}\right)\right]}{logn}$$

where $Y_{j}=\sum_{j=1}^{n}{\tilde{y}}_{ij}$. Note that the value of DEF is a reasonable measurement of the differentially expressed genes. For example, gene *i* is expressed across all samples with reads count 1 after normalization, that is ${\tilde{y}}_{i1}={\tilde{y}}_{i2}=\cdots ={\tilde{y}}_{in}=1$, then

(4) $$H=1-\frac{-\sum_{j=1}^{n}\left[\left(\frac{1}{n}\right)\cdot log\left(\frac{1}{n}\right)\right]}{logn}=1-\frac{-log(\frac{1}{n})}{logn}=0$$

which shows gene *i* is not differentially expressed by the differential entropy-like function. This agrees with the fact gene *i* is not differentially expressed across the samples. For another example, gene *i* is expressed differentially with read counts 1, 2, 3 after normalization across three samples. Then,

(5) $$H=1-\frac{-\frac{1}{6}\times log\frac{1}{6}-\frac{2}{6}\times log\frac{2}{6}-\frac{3}{6}\times log\frac{3}{6}}{log3}=0.079$$

which shows the difference exists across these three samples. The bigger the value *H* is, the greater the expression difference among *n* samples is. For each gene, we calculated a differential entropy-like function value, which permits the direct estimation of the degree of expression. For each gene, its difference degree across multiple samples can be quantified by DEF value. This gene was defined as a differential expression when *H* was larger than a reasonable threshold; otherwise, it was judged as non-differential expression gene. We implemented the method presented in this article in the software package DEF, which was written in R language. A software implementation is available from https://github.com/xiaoxiaoxier/DEF. DEF expects a matrix of unnormalized count data as input and the output of the analysis is the IDs of the differentially expressed genes. When using DESeq2, edgeR and bayseq for comparison, all parameters were left at their default values. For baySeq, we took 5000 samples for estimating the priors with the quasi-likelihood approach.

## Figures and Tables

**Table 1:** Read counts of top five unique differentially expressed genes detected by DEF in “Sultan” dataset.

Ensembl ID	Condition A	Replicate 2	Condition B	Replicate 2	DEF Value
	Replicate 1		Replicate 1		
ENSG00000164002	5	0	0	0	0.0617
ENSG00000104833	0	0	7	0	0.0608
ENSG00000124920	0	0	7	0	0.0608
ENSG00000182310	0	0	7	0	0.0608
ENSG00000197608	0	0	0	6	0.0581

**Table 2:** Read counts of last five unique differentially expressed genes detected by DEF in “Sultan” dataset.

Ensembl ID	Condition A	Replicate 2	Condition B	Replicate 2	DEF Value
	Replicate 1		Replicate 1		
ENSG00000111325	4	8	6	2	0.0101
ENSG00000141431	1	1	5	4	0.0100
ENSG00000065357	4	10	3	4	0.0100
ENSG00000179021	2	6	6	2	0.0100
ENSG00000215301	2	6	6	2	00100

**Table 3:** Read counts of top five unique differentially expressed genes detected by DEF in “Katz” dataset.

Ensembl ID	Condition A	Replicate 2	Condition B	Replicate 2	DEF Value
	Replicate 1		Replicate 1		
ENSMUSG00000051920	0	0	5	0	0.0437
ENSMUSG00000029683	0	0	0	4	0.0436
ENSMUSG00000069301	0	0	0	4	0.0436
ENSMUSG00000070691	0	0	1	4	0.0432
ENSMUSG00000079332	0	0	3	2	0.0412

**Table 4:** Read counts of last five unique differentially expressed genes detected by DEF in “Katz” dataset.

Ensembl ID	Condition A	Replicate 2	Condition B	Replicate 2	DEF Value
	Replicate 1		Replicate 1		
ENSMUSG00000038593	6	3	4	0	0.0101
ENSMUSG00000036977	5	11	0	1	0.0101
ENSMUSG00000057924	2	2	5	1	0.0101
ENSMUSG00000067203	2	2	5	1	0.0101
ENSMUSG00000002205	2	13	10	3	0.0100

**Table 5:** Top five differentially expressed genes obtained from DEF.

Ensembl ID	Gene Symbol	Gene Function
ENSMUSG00000051617	Krt9	An important special function in the mature palmar
		Plays an essential role in the correct development of sperm
ENSMUSG00000020123	Avpr1a	Receptor for arginine vasopressin
ENSMUSG00000050505	Pcdh20	Potential calcium-dependent cell-adhesion protein.
ENSMUSG00000027919	Lce1g	keratinocyte differentiation
ENSMUSG00000045903	Npas4	A key role in the structural and functional plasticity of neurons
		Transcription factor expressed in neurons of the brain

**Table 6:** Top five differentially expressed genes obtained from DEF in the “Cheung” dataset.

Ensembl ID	Gene Symbol	Gene Function
ENSG00000204644	ZFP57	May serve an important special function either in the mature palmar
		Plays an essential role in the correct development of sperm
ENSG00000007038	PRSS21	Receptor for arginine vasopressin
ENSG00000179455	MKRN	Potential calcium-dependent cell-adhesion protein.
ENSG00000170627	GTSF1	Protein coding
ENSG00000129824	RPS4Y1	multicellular organism development
		nuclear-transcribed mRNA catabolic process, nonsense-mediated decay

**Table 7:** Top five differentially expressed genes obtained from DEF in the “Wang” dataset.

Ensembl ID	Gene Symbol	Gene Function
ENSG00000100721	TCL1A	Enhances cell proliferation, stabilizes mitochondrial membrane potential and promotes cell survival
		Enhances the phosphorylation and activation of AKT1, AKT2 and AKT3.
ENSG00000110777	POU2AF1	It is essential for the response of B-cells to antigens and required for the formation of germinal centers
ENSG00000111348	ARHGDIB	Regulates the GDP/GTP exchange reaction of the Rho proteins by inhibiting the dissociation of GDP from them, and the subsequent binding of GTP to them
ENSG00000118308	LRMP	Plays a role in the delivery of peptides to major histocompatibility complex (MHC) class I molecules
		May play a role during fertilization in pronucleus congression and fusion
ENSG00000133124	IRS4	Acts as an interface between multiple growth factor receptors possessing tyrosine kinase activity
		Plays a pivotal role in the proliferation/differentiation of hepatoblastoma cell
		Plays a role in growth, reproduction and glucose homeostasis

**Table 8:** Five datasets information.

Abbreviate in the Article	Number of Samples	Note
Sultan (human) [40]	4	cell type comparison
Katz (mouse) [41]	4	case and control comparison
Trappnel l(mouse) [2]	4	time course comparison
Cheung (human) [30]	41	individual comparison
Wang (human) [42]	9	tissue comparison

## Abbreviations

The following abbreviations are used in this manuscript:

DEF	Differential Entropy-like Function
NGS	Next Generation Sequencing
DEG	Differentially Expressed Gene
